# Are different groups of patients with stroke more likely to be excluded from the new UK general medical services contract? A cross-sectional retrospective analysis of a large primary care population

**DOI:** 10.1186/1471-2296-8-56

**Published:** 2007-09-27

**Authors:** Colin R Simpson, Philip C Hannaford, Matthew McGovern, Michael W Taylor, Paul N Green, Karen Lefevre, David J Williams

**Affiliations:** 1 Department of General Practice and Primary Care, Foresterhill Health Centre, Westburn Road, The University of Aberdeen, Aberdeen, AB25 2AY, UK; 2 Kemnay Medical Group, Kemnay, Aberdeenshire, UK; 3 Department of Clinical Pharmacology, Grampian Universities Hospital Trust, Aberdeen, UK

## Abstract

**Background:**

In April 2004, an incentive based contract was introduced to UK primary care. An important element of the new contract is the ability to exclude individuals from quality indicators for a variety of reasons (known as 'exception reporting'). Exception of patients with stroke or TIA from the recording and achievement of quality indicators may have important consequences in terms of stroke recurrence and mortality.

**Methods:**

A cross-sectional retrospective analysis of anonymised patient data was performed using 312 Scottish primary care practices.

**Results:**

Patients recorded as unsuitable for inclusion in the contract were more likely to be female (odds ratio (OR) 1.51, 95% confidence interval (CI) 1.36–1.68), older (>75 years:OR 3.15, 95%CI 2.69–3.69), and have dementia (OR 4.40, 95%CI 3.57–5.43) when compared to those patients without such a code. Patients were less likely to be older (>75 years:OR 0.70, 95%CI 0.56–0.87) and were more likely to be from the most deprived areas of Scotland (Quintile 5: OR 2.02, 95%CI 1.50–2.70) if they refused to attend for review or did not reply to letters asking for attendance at primary care clinics. Patients with multiple co-morbidities were more likely to have exclusions for achieving diagnostic clinical targets such as cholesterol control (3 or more co-morbidities: OR 3.37, 95%CI 2.50–4.50).

**Conclusion:**

Scottish practices have appeared to use exception reporting appropriately by excluding patients who are older or have dementia. However, younger or more socio-economically deprived patients were more likely to be recorded as having refused to attend for review or not replying to letters asking for attendance at primary care clinics. It is important for primary care practices to identify and monitor these individuals so that all patients fully benefit from the implementation of an incentive based contract and receive appropriate clinical care to prevent stroke recurrence, further disability and mortality.

## Background

Virtually all individuals resident in Scotland (including children) are registered with primary care, which is free at the point of contact and provides first line and continuing post-hospitalisation care of patients. Access to secondary care is usually obtained through a primary care (general) practice and even if a patient is admitted to hospital (e.g. because of an emergency) details of the stay are reported back to the patient's primary care practice. In April 2004, a new quality-based General Medical Services (nGMS) contract was introduced to UK primary care, which reduced the proportion of income of general practitioners (GP) derived from per capita payments and increased the proportion (approximately 23%) derived from providing specific aspects of care, such as targets based on quality indicators [1]. The new contract provides payments to practices to develop an accurate register of patients who have had a stroke (since a complete and accurate register is a prerequisite for monitoring patients) and for the recording of smoking habits, blood pressure and cholesterol levels of patients on the register. Further payments are paid for reaching a number of specific treatment targets, for example blood pressure control [1]. An analysis of aggregated data collated from almost all UK general practices, revealed that practices earned an average of £76,200 (USD $142,120) from meeting quality related targets in the nGMS contract [2]. Scotland has the highest mortality rate for stroke in Western Europe [3] and therefore it was reassuring that the recording of quality indicators directly relating to stroke or transient ischaemic attack (TIA) care were found to have increased by an average of 40% in the year after the introduction of the contract [4]. However, there were differences in the recording and ascertainment of blood pressure and cholesterol measurements. There were also differences in secondary preventative prescribing between groups of patients such as women, the elderly and the most deprived.

An important element of the new contract is the ability to exempt individuals from the recording of quality indicators for a variety of reasons (a practice known as 'exception reporting'). In such circumstances, 'ineligible patients' are removed from the denominator of that quality indicator, so that a practice's ability to reach different thresholds for payment is not adversely affected. Patients may be excepted from all indicators relating to a clinical domain, for example, if individuals with stroke/TIA are too frail, refuse to attend for review or do not reply to letters asking for attendance at primary care clinics; known as 'top level' exceptions [5].

Practices that contributed data to the quality management and analysis system (QMAS), a national information technology system in the UK and performed well in achieving national Quality and Outcomes Framework achievement targets were found to have excluded large numbers of patients by exception reporting, with one percent of practices excluding 15 percent of their patients [2]. It was hypothesised by Doran and colleagues that practices that were better at identifying and treating patients with chronic conditions also tended to identify more patients for whom the targets were inappropriate [2]. The median exclusion rate found for practices contributing to QMAS was 6.1%. Practices with lower levels of exception reporting tended to have larger proportions of elderly patients (p < 0.01) and patients without any formal educational qualifications (p < 0.05). More patients were excluded if they were registered as having conditions such as stroke/TIA (median rate: 6.5%, interquartile range (IQR) 3.8% to 9.0%) or coronary heart disease (median rate 7.8%. IQR 5.4% to 10.4%). Fewer patients were excluded if they were registered as having hypertension (median rate 0.9%, IQR 0.5% to 1.7%) or hypothyroidism (median rate 0.8%, IQR 0.0% to 2.0%).

Further analysis of practices in Scotland found that significant variation occurred in the use of exception reporting amongst practices across all disease domains [6], with deprivation differences found in the delivery of quality of certain indicators such as diagnostic procedures (blood pressure, cholesterol) [7]. Deprived primary care practices in the Brighton and Hove area of England were more likely to use exception reporting for diabetes quality indicators [8]. These studies have used publicly available national datasets such as QMAS to identify the characteristics of practices with high levels of exception reporting [2,6-8]. However, these datasets do not provide access to individual patient level data and therefore cannot be used to determine the individual characteristics of excluded patients. Exception of patients with stroke or TIA from the recording and achievement of quality indicators may have important consequences in terms of stroke recurrence and mortality. We therefore used electronic data from primary care practices in Scotland available to PCCIU to identify whether certain groups of patients were more likely to be recorded with exception coding.

## Methods

Anonymous computerised clinical data from the 310 of 1031 Scottish practices that participate in Scottish Programme for Improving Clinical Effectiveness (SPICE), part of a Clinical Effectiveness Programme developed by the Royal College of General Practitioners (Scotland) [9]. Although self-selected, these practices are broadly representative of the Scottish population and care for 1,775,397 of 5,094,800 patients (34.8%)[10].

From these data, we examined individuals who had a computer record of stroke or TIA. Stroke/TIA patients eligible for exclusion by practices from the stroke clinical domain using the 'top level' exception reporting codes (overall stroke exception reporting, patient unsuitable (e.g. extreme frailty), informed dissent (refusing to attend for review) or no response to letters to attend to a clinic) were identified. In addition, patients eligible for exclusion from individual quality indicators were also identified. The specific quality indicators analysed included: achieving blood pressure (≤150 mmHg systolic and ≤90 mmHg diastolic) or cholesterol (≤5 mmol/l) control and receiving antiplatelets/anticoagulants or influenza vaccination if they were on maximum tolerated therapy, refusal to undergo a procedure or had a contraindication, adverse or allergic reactions to therapy. Key characteristics for these patients were determined including: sex; age (<64, 65 to 75 or 75+ years); number of stroke-related co-morbidities, (hypertension, atrial fibrillation, coronary heart disease, heart failure and peripheral vascular disease: 0, 1, 2 or 3+); dementia, and area measure of deprivation (based on the Carstairs measure of deprivation derived from postcode: deprivation quintile 1 being the least and quintile 5 the most deprived [11]) all as pertaining on 1^st ^April 2005.

### Statistical analysis

To examine the association between stroke/TIA prevalence and frequency of recording of 'top level' exception codes, the Spearman correlation coefficient was calculated. Binary logistic regression was used to determine odds ratios (OR) and 95% confidence intervals (95% CI) for the recording of exception codes among different gender, age, deprivation, co-morbidity and dementia groups, adjusted for potential confounding by the same factors (except where a factor was itself being explored). Adjustment was also made to take into account clustering of patients within practices.

For clarity, all proportions are presented to one decimal place. All analyses were performed using SPSS for Windows 14.0 (SPSS inc, Chicago, Illinois, USA) and STATA 9.2 (Statacorp, College Station, Texas, USA). The study protocol was approved by the Scientific Advisory Group of the Primary Care Clinical Informatics Unit.

## Results

In April 2005, 32,401 patients were recorded as having had a stroke/TIA in the participating SPICE practices. The median prevalence per practice was 1.8% (interquartile range 1.5% to 2.2%). This stroke/TIA population has been described previously [4].

108 (34.6%) practices did not have any stroke/TIA patients with 'top level' exception codes. Practice recording of 'top level' exception codes was not associated with the practice having proportionately more female, older or deprived stroke/TIA patients (table 1). However, there was a weak but significant association between practice exception reporting and the number of stroke/TIA patients with co-morbidities and also the proportion of the overall practice population registered as having stroke or TIA. The median percentage of patients with 'top level' exception codes found among all study practices was 7.0% (interquartile range of 3.1% to 13.8%).

**Table 1:** Spearman correlation coefficients for practice characteristics and exception reporting.

	**Correlation Coefficient**	**P value**
**Gender**	0.00	0.97
**Age (in years)**	0.08	0.15
**Co-morbidity**	0.42	<0.001
**Deprivation**	0.10	0.08
**Stroke/TIA population size**	0.18	<0.05

1,749 individuals with stroke/TIA from SPICE practices had the 'patient unsuitable' exception reporting code (table 2). Stroke/TIA patients with this 'top level' exclusion code were more likely to be female, older, and have a diagnosis of dementia when compared to those patients without such a code. 295 patients with stroke/TIA had the 'top level' 'informed dissent' and 154 the 'no response to letter' code (table 2).

**Table 2:** Characteristics of stroke/transient ischaemic attack patients recorded with clinical domain 'top level' exception codes

	**Unsuitable for inclusion n = 1749**		**Refusal or no response to letters to attend clinic n = 449**	
	**n (% Stroke)**	**Odds ratio (95% CI)**	**n (% Stroke)**	**Odds ratio (95% CI)**
**Gender:**				
Men*	623 (3.9)	1.00	218 (1.3)	1.00
Women	1126 (7.0)	1.51 (1.36–1.68)	231 (1.4)	1.07 (0.89–1.29)
**Ageband**:				
<65*	189 (2.4)	1.00	150 (1.9)	1.00
65–74	257 (2.7)	1.13 (0.93–1.37)	105 (1.1)	0.60 (0.46–0.77)
>75	1303 (8.7)	3.15 (2.69–3.69)	194 (1.3)	0.70 (0.56–0.87)
**Deprivation:**				
Quintile 1*	362 (5.8)	1.00	76 (1.2)	1.00
Quintile 2	391 (5.6)	1.03 (0.88–1.19)	72 (1.0)	0.83 (0.60–1.15)
Quintile 3	414 (5.8)	1.05 (0.90–1.21)	89 (1.2)	1.00 (0.73–1.36)
Quintile 4	324 (4.5)	0.84 (0.71–0.98)	95 (1.3)	1.08 (0.80–1.46)
Quintile 5	258 (5.5)	1.15 (0.97–1.36)	117 (2.5)	2.02 (1.50–2.70)
**Co-morbidities:**				
0*†	529 (4.8)	1.00	140 (1.3)	1.00
1	614 (5.8)	1.08 (0.96–1.22)	171 (1.6)	1.29 (1.03–1.62)
2	338 (5.1)	0.91 (0.79–1.05)	86 (1.3)	1.08 (0.82–1.42)
3+	268 (6.5)	1.14 (0.97–1.33)	52 (1.3)	1.06 (0.76–1.46)
**Dementia:**				
No*	1474 (4.7)	1.00	433 (1.4)	1.00
Yes	275 (28.0)	5.32 (4.56–6.20)	16 (1.6)	1.25 (0.75–2.09)

*Reference group for odds ratio

† Stroke-related co-morbidity including diabetes, hypertension, atrial fibrillation, coronary heart disease, heart failure and peripheral vascular disease.

The youngest and most deprived groups of patients were more likely to be exempted from the contract for these reasons. 198 patients were coded as having been prescribed maximum tolerated blood pressure therapy and were eligible for exclusion from achieving blood pressure control (table 3). Female patients and those with one or more co-morbidities were more likely to receive these codes with dementia patients being less likely. 605 patients were coded as having been prescribed maximum lipid lowering therapy or having adverse/allergic reaction to a statin and were eligible for exclusion from achieving cholesterol control (table 3). Female patients and those with one or more co-morbidities were more likely to receive these codes. Thirty nine patients with TIA or stroke were eligible for exclusion from the influenza immunisation and nine patients with non-haemorrhagic stroke, or a history of TIA were eligible for exclusion from receiving an anti-platelet (aspirin, clopidogrel, dipyridamole), or anti-coagulant (warfarin) agent.

**Table 3:** Characteristics of stroke/transient ischaemic attack patients recorded with individual quality indicator exception codes

	**Excluded from achieving blood pressure control n = 198**		**Excluded from achieving cholesterol control n = 605**	
	**n (% Stroke)**	**Odds ratio (95% CI)**	**n (% Stroke)**	**Odds ratio (95% CI)**
**Gender:**				
Men*	66 (0.4)	1.00	361 (1.9)	1.00
Women	132 (0.7)	2.12 (1.59–2.83)	244 (1.3)	1.56 (1.30–1.88)
**Ageband**:				
<65*	34 (0.3)	1.00	143 (1.4)	1.00
65–74	58 (0.6)	1.11 (0.74–0.66)	210 (2.0)	1.18 (0.92–1.50)
>75	106 (0.6)	1.17 (0.47–1.35)	252 (1.5)	0.83 (0.68–1.01)
**Deprivation:**				
Quintile 1*	39 (0.5)	1.00	115 (1.6)	1.00
Quintile 2	35 (0.4)	0.80 (0.63–1.73)	123 (1.5)	0.95 (0.68–1.31)
Quintile 3	50 (0.6)	1.05 (0.59–1.58)	134 (1.6)	1.10 (0.68–1.37)
Quintile 4	43 (0.5)	0.96 (0.55–1.82)	148 (1.8)	0.93 (0.79–1.53)
Quintile 5	31 (0.6)	1.00 (0.55–1.82)	85 (1.6)	1.00 (0.59–1.44)
**Co-morbidities:**				
0*†	4 (0.03)	1.00	95 (0.7)	1.00
1	64 (0.5)	17.77 (6.41–49.28)	239 (2.0)	2.88 (2.21–3.77)
2	75 (1.0)	35.39 (12.65–98.98)	172 (2.4)	3.54 (2.71–4.63)
3+	55 (1.2)	42.85 (15.02–122.22)	99 (2.2)	3.37 (2.50–4.54)
**Dementia:**				
No*	197 (0.6)	1.00	592 (1.6)	1.00
Yes	1 (0.1)	0.11 (0.02–0.82)	14 (1.3)	0.71 (0.41–1.22)

*Reference group for odds ratio

† Stroke-related co-morbidity including diabetes, hypertension, atrial fibrillation, coronary heart disease, heart failure and peripheral vascular disease.

## Discussion

In this analysis of electronic patient data from primary care, there was evidence of an association between exception reporting and the percentage of all patients registered with stroke/TIA patients, and stroke/TIA patient co-morbidity. Patients with stroke/TIA who were recorded as having a 'top level' exception code: 'unsuitable for inclusion' were more likely to be female, older and be diagnosed with dementia. Younger patients and patients from more deprived parts of Scotland were more likely to have the exception codes: 'informed dissent' or 'no response to letters'. Females and those with one or more comorbidities were more likely to be excluded from the specific quality indicators of achieving blood pressure or cholesterol control. Only a small number of patients were excluded from the influenza vaccine and antiplatelet/anti-coagulant therapy indicators.

The median practice percentage of patients with stroke/TIA (1.8%) was similar to the level (2.2%) found in the 2003 Scottish Health Survey of adults over 16 years of age [12]. The median percentage of stroke/TIA patients with a 'top level' exception reporting code (7.0%), was similar to the level (6.1%) found among the 8,576 primary care practices in the UK that contributed data to QMAS [2]. Our finding that there was no association between practice exception reporting and age and deprivation, did not correspond with the practice level analysis performed using QMAS data which found that practices with older populations were less likely to exclude their patients [2]. Nor did we find deprivation differences in the use of exclusions for certain diagnostic indicators such as blood pressure and cholesterol [8]. Although patients registered with SPICE practices have been shown to be representative of the Scottish population [10], SPICE practices themselves are not fully representative of all Scottish practices as proportionally fewer are single-handed or located in the most deprived areas of Scotland [13].

After the first year of the nGMS contract, practices with a high rate of stroke/TIA were more likely to exclude their patients from the nGMS contract. This finding supports the notion that practices that were better at identifying patients with chronic conditions tended to identify more patients for whom exclusion from the contract was appropriate [2]. Patients with stroke/TIA who were female, older and diagnosed with dementia were more likely to be recorded as 'unsuitable for inclusion'. It is possible that older patients with dementia were frail and therefore these patients may have been given an exception code for clinically valid reasons. The finding that patients with co-morbidities were more likely to be excluded from the achievement of blood pressure or cholesterol targets, may reflect the likelihood that these patients did not tolerate these therapies or received multiple other treatments which may cause adverse events.

## Conclusion

The introduction of the nGMS contract to Scottish primary care has led to a substantial increase in the measurement of quality indicators and secondary preventative treatment of patients with prior stroke or TIA [4]. These practices have appeared to use exception reporting appropriately by excluding patients who are older or have dementia. However, younger or more socio-economically deprived patients were more likely to be recorded as having refused to attend for review or not replying to letters asking for attendance at primary care clinics. It is important for primary care practices to identify and monitor these individuals so that all patients fully benefit from the implementation of an incentive based contract and receive appropriate clinical care to prevent stroke recurrence, further disability and mortality.

## Competing interests

The author(s) declare that they have no competing interests.

## Authors' contributions

The planning of the study was undertaken by all the authors. The data were extracted, analysed and the paper was written by CS. All authors have contributed to end-drafts and approved the final manuscript.

## Pre-publication history

The pre-publication history for this paper can be accessed here:


